# VR sculpting as a therapeutic intervention for alleviating anxiety: a case study from a university art class

**DOI:** 10.3389/fpsyt.2025.1588745

**Published:** 2025-05-29

**Authors:** Huan Ding, Bo Lin, Yuwei Xu, Dunjiang Shang, Yi Han

**Affiliations:** ^1^ School of Design and Innovation, Xiamen University Tan Kah Kee College, Zhangzhou, China; ^2^ Faculty of Humanities and Arts, Macau University of Science and Technology, Macao, Macao SAR, China; ^3^ Institute of Education, Xiamen University, Xiamen, China

**Keywords:** virtual reality sculpting, anxiety reduction, higher education, art therapy intervention, art classes

## Abstract

**Background:**

Anxiety among university students has become increasingly prominent in higher education settings, adversely affecting not only their academic performance but also their overall mental health. While medication and psychological interventions can help alleviate anxiety to some extent, it is crucial for this particular group to find more convenient, easily accepted non-pharmacological approaches. Art therapy, especially hands-on creative activities, is considered an effective means of regulating anxiety, yet research on virtual reality (VR) sculpting remains relatively scarce.

**Objective:**

This paper aims to investigate the therapeutic effects of VR sculpting on college students’ anxiety levels and assess its impact on psychological well-being and physiological relaxation, as measured by heart rate variability (HRV).

**Methods:**

The study recruited 30 undergraduates, evaluating their anxiety levels via the Generalized Anxiety Disorder scale (GAD-7), psychological well-being via the Positive and Negative Affect Schedule (PANAS), and physiological relaxation via HRV. Subsequently, we monitored how VR-based artistic sculpting influenced changes in GAD-7, PANAS, and HRV scores.

**Results:**

Comparing the data before and after the art-based intervention revealed a significant decrease in participants’ GAD-7 scores, a substantial rise in PANAS scores indicating enhanced psychological well-being (p < 0.05), and a marked increase in HRV, suggesting VR sculpting can promote physiological relaxation. Qualitative interviews further showed that most participants experienced strong focus and emotional release during the VR sculpting process.

**Conclusion:**

As a non-pharmacological art therapy method, VR sculpting demonstrates a notably positive effect on reducing anxiety and improving mental well-being among university students. This study provides compelling evidence for using art therapy to address mental health issues in a college environment. Future research should consider longer intervention periods and more varied measures to further investigate the long-term effectiveness and broader applicability of this approach.

## 1 Introduction

In recent years, anxiety among college students has become increasingly prominent, posing a key factor affecting their academic performance, social adaptation, career planning, and overall mental health. According to the World Health Organization (WHO) and multiple international surveys, the prevalence of anxiety symptoms in university populations continues to rise (1). One study revealed that major contributors to college students’ anxiety include academic pressure, career uncertainty, and tense interpersonal relationships, with significant differences in anxiety levels associated with gender, year of study, and family background (2). Moreover, anxiety not only undermines students’ concentration and learning efficiency but is also closely tied to sleep disturbances, low self-esteem, and strained social interactions (3). Prolonged high levels of anxiety can lead to concentration problems and weakened logical thinking, potentially triggering more serious mental health issues such as chronic insomnia, depressive symptoms, and even an increased risk of self-harm or dropout (4). Consequently, research on anxiety in college students holds considerable practical importance, helping to devise effective interventions that reduce the long-term adverse effects of anxiety on student mental health.

Among existing interventions for college student anxiety, medication and psychological counseling are the two primary approaches. Medication (e.g., anti-anxiety drugs) can provide short-term relief for certain anxiety symptoms, but its side effects, potential for dependence, and associated social stigma often lead many students to harbor concerns about pharmacological treatments (5). Psychological counseling—such as cognitive behavioral therapy (CBT), psychodynamic therapy, and interpersonal therapy—possesses relatively robust theoretical and practical foundations. However, in university settings, factors like limited availability of professional counselors, student resistance to “talk-based” therapy, as well as cultural and environmental stigma, continue to hinder its widespread adoption (6). In recent years, non-pharmacological interventions have increasingly gained attention. For instance, internet-based cognitive behavioral therapy (CBT) has been shown to significantly improve anxiety symptoms among undergraduates. One study found that participation in online group CBT significantly reduced both anxiety and depression, with high acceptability and convenience (7). Furthermore, interventions like art therapy have also been found to effectively lower anxiety levels in college students and enhance their emotional regulation skills (8). Hence, identifying a non-pharmacological intervention that is easy to implement, cost-effective, widely acceptable, and delivers marked therapeutic benefits has become a pressing need in university mental health education and management.

In this context, many scholars have recently turned to “Art Therapy,” aiming to address issues of anxiety and depression through non-traditional therapeutic means. Studies have indicated that art therapy can effectively reduce anxiety levels in college students and increase their capacity to regulate emotions (9). Through various forms—such as painting, music, dance, and sculpting—art therapy helps individuals express their inner emotions and hidden conflicts, engaging multiple senses in the process to foster emotional release and regulation (10). Compared with traditional verbal expression, art therapy highlights a holistic mind-body experience, allowing individuals to channel and release emotions through visual, auditory, tactile, and even kinesthetic avenues. Research shows that mandala-based art therapy interventions for undergraduates significantly alleviate anxiety, depression, and stress while maintaining high acceptability (11). Moreover, personalized art interventions (e.g., expressive arts therapy) similarly show strong effectiveness in easing anxiety (12). For college students, art-based interventions not only help diminish the stigma surrounding “mental illness” or “counseling,” but also offer a more discreet and acceptable means of self-healing for those who might hesitate to seek formal treatment (13).

However, most existing art therapy research focuses on painting, music, or dance, which have each demonstrated positive effects in reducing anxiety, depression, and stress. Compared with these established art forms, sculpting has gradually garnered scholarly interest due to its tangible, hands-on nature. The repetitive interaction with materials in sculpting compels individuals to focus on texture, shape, and structure, fostering deep concentration and self-exploration during the “hands-on–perception–shaping” process (14). Moreover, sculpting can heighten one’s sense of control over reality and serve as an alternative channel for emotional expression, granting psychological satisfaction and relaxation through the creative process (15).

In recent years, virtual reality (VR) has demonstrated remarkable effects in mitigating anxiety and depression within mental health fields (16), offering new opportunities for art therapy. Particularly in sculpting, VR-based creation has emerged as a valuable technological intervention. By incorporating virtual reality, VR sculpting transcends the spatial and material limitations of traditional sculpting, enabling individuals to carry out sculptural creation in a virtual world. This method not only simulates the real process of sculpting but, through immersive experiences provided by visual and tactile feedback, significantly elevates emotional engagement and creative focus (17). Typically, VR sculpting employs specialized VR headsets, handheld controllers, sensors, and software that permit interactive modeling. Upon entering the virtual 3D environment, participants use virtual tools and materials to sculpt. The VR system offers precise feedback: in addition to visual immersion from the headset, haptic vibrations and force feedback from the controllers replicate the tactile sensation of genuine sculpting, making users feel as though they are operating in a digitally constructed environment (18). The immediate visual feedback and motion tracking improve the creator’s attention to detail and stimulate creativity (19). Overall, the immersive experience in VR can help enhance concentration, reduce anxiety levels, and, because of its high acceptability among younger users, could be more effectively introduced to the university population (20).

VR sculpting, as a new mode of art therapy, has seen a rising number of studies highlighting its potential for emotional healing. Firstly, VR sculpting alleviates anxiety and stress by enabling participants to express and release emotions during creation (21). Sculpting, in essence, has a pronounced physical component, and while engaged in VR sculpting, participants must maintain intense focus, shaping virtual materials and tools in real time. The deep involvement allows individuals to externalize negative feelings through hands-on creative activities, easing internal tension and anxiety (22). Secondly, VR sculpting promotes a flow experience, which aids in enhancing mood and promoting a highly relaxed state (20). Research shows that immersion in virtual environments for sculpting substantially reduces psychological pressure and anxiety while eliciting feelings of pleasure and satisfaction, similar to the emotional benefits found in traditional sculpting (23). Lastly, VR-based sculpting offers more freedom and scope for exploration. Users can select from a range of tools and materials without the physical constraints and technical barriers commonly found in real-world sculpting, devoting more energy to emotional expression and self-discovery (24). Compared with physical sculpting, VR sculpting is more flexible, simpler to operate, and avoids potential safety hazards. It also allows more immediate feedback aligned with the user’s psychological state—particularly beneficial for psychological counseling and emotional regulation (25).

Several studies investigating the physiological effects of VR sculpting interventions have emphasized heart rate variability (HRV) as a key indicator of autonomic nervous system function and stress response. Studies suggest VR-based art therapy can effectively raise participants’ HRV levels, placing them in a more relaxed state and improving physiological resilience to stress (26). VR coupled with HRV biofeedback (HRV-BF) has proven especially effective for lowering stress and helping individuals better manage autonomic responses (27). Participants engaging in stress-management training within VR environments show significantly increased HRV along with notably reduced anxiety and stress (28). Therefore, VR sculpting may offer preliminary benefits in reducing anxiety and supporting physiological adaptation, though further validation is needed to assess its long-term effects on stress resilience (29). This finding underscores the extensive potential for VR-based immersion in mental health interventions, offering new directions for VR-focused art therapy research (30).

Despite preliminary evidence suggesting the benefits of VR-based art therapy—specifically VR sculpting—in reducing anxiety, improving emotional states, and boosting mental well-being, systematic research targeting college students remains limited. Key issues yet to be conclusively examined include effectively implementing VR sculpting interventions for college populations, selecting suitable materials and techniques to maximize immersion and concentration, and clarifying VR sculpting’s impact on psychological and physiological indices. Employing a repeated-measures experimental design and combining psychological scales, HRV monitoring, and qualitative interviews, this study seeks to investigate the effectiveness of VR sculpting interventions for undergraduates. We aim to address the method’s efficacy in alleviating anxiety, enhancing psychological well-being, and promoting physiological relaxation, thereby offering practical and scalable art-based mental health strategies for university settings.

## 2 Methods

### 2.1 Study design

This study adopted a repeated-measures design, wherein the same group of participants underwent both control and experimental interventions as well as measurements. The aim was to more precisely evaluate the intervention’s effectiveness and to minimize confounding influences due to individual differences. In the field of psychological intervention research, repeated-measures designs typically demonstrate strong statistical power and help control for individual variability, thereby enhancing the internal validity of the study (31). The specific procedures were as follows.

#### 2.1.1 Control phase

During this phase, researchers collected baseline data on participants’ anxiety (via GAD-7), emotional affect (via PANAS), and physiological state (via HRV), while participants continued their usual academic and daily routines. No art-based intervention or counseling was introduced at this stage, ensuring a control condition for comparison. In the experimental phase, the same group participated in a VR sculpting session. Before the session, baseline data were collected again. After the session, participants completed the same GAD-7 and PANAS assessments, and HRV data were recorded again. This repeated-measures structure allowed for within-subject comparisons.

#### 2.1.2 Experimental phase

The same group of participants received a “VR sculpting” intervention, completing the creative process within a set time frame and specific environment (a virtual reality system). Immediately following the intervention, various measures (such as GAD-7, PANAS, and HRV) were assessed and recorded.

### 2.2 Participants

This study recruited 30 undergraduates from a university in Xiamen, all of whom were juniors or seniors (ages 21–23). Each volunteer provided informed consent before joining the research. During recruitment, the researchers explained the objectives, potential risks, and expected benefits of the study, ensuring that all participants met the following criteria: (1) physically and psychologically healthy, with no serious physical illnesses or diagnosed mental disorders; (2) no recent participation in similar interventions, specifically no involvement in systematic art therapy or other psychological interventions; and (3) no strong aversion to artistic activities, and at least a basic interest or willingness to try VR sculpting. Participants were informed that they could withdraw from the study at any time, without any negative consequences for their academic standing or personal rights.

### 2.3 Interventions

#### 2.3.1 Venue and environment

To ensure immersion and safety, the intervention was conducted in a dedicated VR-equipped laboratory or specialized VR classroom on campus. The space was relatively quiet, provided suitable lighting, and offered ample room for movement while wearing VR devices, thereby minimizing the risk of collisions or injuries.

#### 2.3.2 Materials and equipment preparation

The VR setup used for this study was the Oculus Quest 2 with haptic feedback controllers. This system was selected based on its accessibility, immersive performance, and safety profile for educational interventions. Regarding VR hardware, a head-mounted display (HMD) and handheld controllers or force-feedback devices with robust three-dimensional tracking capabilities were provided to accurately capture the user’s hand movements and positions. As for the VR sculpting software, a 3D creation platform simulating realistic sculpting processes was employed, supporting a range of virtual materials (e.g., wood, stone, clay) and tools (e.g., chisels, scrapers, brushes), along with real-time rendering of different carving effects or texture changes. Prior to each session, the researcher inspected the VR equipment and environment, advised participants on safe movement boundaries, and offered technical guidance to minimize dizziness or discomfort.

#### 2.3.3 Creative process

Before participants began, the researcher spent five minutes demonstrating basic VR system operations, tool switching, and safety considerations. Participants then donned VR headsets to start freeform creation, selecting virtual tools and materials that suited their interests or current emotional state for 20 to 30 minutes. The researcher minimized specific thematic or stylistic guidance, intervening only when necessary to provide technical support or safety alerts. Through real-time immersive visual and haptic feedback, participants could concentrate on their work in a three-dimensional space, achieving both emotional expression and relaxation. Once creation concluded, participants could save or exhibit their pieces within the VR software, and also had the option to display a preview of the finished product on a shared screen. The researcher or other team members might pose simple questions or offer encouraging comments, prompting participants to reflect on their emotional experiences and personal impressions during creation. This VR sculpting intervention aimed to immerse students in a deeply engaging environment, allowing them to focus on “hands-on” and “flow” experiences, thereby facilitating emotional release and psychological adjustment.

### 2.4 Measurements

In this study, the Generalized Anxiety Disorder-7 (GAD-7, see **Table 1**) scale was used to assess participants’ anxiety symptoms—such as tension, fear, and worry—over the past two weeks. GAD-7 is widely utilized in clinical and research contexts due to its strong reliability and validity, and has been validated across diverse populations (32). Within the scope of this research, GAD-7 enables the objective quantification of how VR sculpting interventions influence anxiety levels and furnishes reliable longitudinal measurements. Each item is rated on a 1 to 4 scale, with higher scores indicating more severe anxiety.

**Table 1:** Generalized anxiety disorder-7.

Questions	Not at all (0)	Several days (1)	More than the average number of days (2)	Almost every day (3)
1. Feeling nervous, anxious, or “on the edge”?				
2. Can’t stop or control worrying?				
3. Worrying too much about everything?				
4. Having trouble relaxing?				
5. Having trouble sitting still because of restlessness or instability?				
6. Irritable or irritable?				
7. Feeling scared, as if something terrible is about to happen?				

The total score is obtained by summing the scores for all seven items. The scoring range is as follows:

0–4 points: Minimal or no anxiety; 5–9 points: Mild anxiety; 10–14 points: Moderate anxiety; 15–21 points: Severe anxiety.

In this study, the Positive and Negative Affect Schedule (PANAS, see **Tables 2**, **3**) was employed to evaluate participants’ emotional states. PANAS comprises two dimensions—Positive Affect (PA) and Negative Affect (NA)—which respectively gauge an individual’s positive and negative emotions. The PA subscale effectively measures one’s positive emotional experiences, while the NA subscale reflects negative emotional states closely associated with anxiety and depression symptoms (33). Each subscale contains ten adjectives, and participants are asked to self-rate each on a five-point scale. The combined scores provide an indication of changes in psychological well-being.

**Table 2:** Positive affect schedule.

Questions	Not at all (1)	Rarely (2)	Sometimes (3)	Often (4)	Always (5)
1.Feel energized					
2. Get excited					
3. Feel strong					
4. Be proud					
5. Feel passionate					
6. Feel spiritual					
7. Feel focused					
8. Feel happy					
9. Get interested					
10. Be amazed					

The total score for Positive Affect is obtained by summing all item scores within the positive affect subscale.

**Table 3:** Negative affect schedule.

Questions	Not at all (1)	Rarely (2)	Sometimes (3)	Often (4)	Always (5)
1. Feel scared					
2. Feel angry					
3. Feel guilty					
4. Feel upset					
5. Feel pain					
6. Feel ashamed					
7. Feel hostile					
8. Feel contempt					
9. Feel nervous					
10. Feel anxious					

The total score for Negative Affect is derived by summing all item scores within the negative affect subscale.

#### 2.4.1 Physiological indicators

In this study, portable heart rate monitors were used to record the heart rate variability (HRV) parameters of the participants when they were sitting quietly. HRV records using the Polar H10 heart rate sensor, which is a medically certified device. HRV is an important physiological indicator for evaluating the function of the autonomic nervous system (ANS) and an individual’s ability to cope with stress, and has been widely used in mental health and stress-related research (34). The key points of HRV analysis are standard time-domain indicators (such as SDNN), high frequency (HF), and the LF/HF ratio in the frequency domain, which are established markers of the regulation of the autonomic nervous system. All data were processed using Kubios HRV software (standard version) and underwent intermediate manual correction. During the control and experimental stages, multiple measurements were made and the average values were taken for comparative analysis.

### 2.5 Data analysis

The study employed quantitative data analysis, first conducting normality tests on the sample data. For normally distributed data, a paired sample t-test was performed, while for non-normally distributed data, a Wilcoxon signed-rank test was applied (35). Based on the analysis results, differences in GAD-7, PANAS, and HRV data between the control and experimental phases were compared to examine the significance of the VR sculpting intervention.

### 2.6 Ethics and quality control

All procedures in this study conformed to relevant ethical guidelines, and participants’ personal information was kept strictly confidential. In 102 hours of data collection and processing, a two-person verification system or automated management tools were employed to ensure data quality and accuracy.

## 3 Results

The VR sculpting process promoted the physical and mental well-being of university students. In this study, data entry was conducted using EpiData, and data analysis was performed with SPSS 24.0. The intervention effect was evaluated by analyzing the differences between the pre-test and post-test. After testing, GAD-7 (control vs. experimental) met the normality assumption and was analyzed using a paired sample t-test; PANAS+ (control vs. experimental) also met the normality assumption and was analyzed using a paired sample t-test; PANAS- (control vs. experimental) did not meet the normality assumption and was analyzed using the Wilcoxon signed-rank test.

### 3.1 Overall data distribution and description statistics

A total of 30 university students participated in the study, with measurements taken during the control phase (no intervention) and the experimental phase (VR sculpting intervention). The measured variables included anxiety levels (GAD-7), positive affect (PANAS+), negative affect (PANAS-), and heart rate variability (HRV), with mean (M) and standard deviation (SD) calculated for each variable. **Table 4**. summarizes the primary measurements from the control and experimental phases. GAD-7 significantly decreased in the experimental phase, indicating that the VR sculpting intervention had a significant effect on reducing anxiety (t = 7.19, p < 0.001). PANAS+ significantly increased in the experimental phase, suggesting that the VR sculpting intervention effectively enhanced psychological well-being (t = -9.04, p < 0.001). PANAS- significantly decreased in the experimental phase, demonstrating that the VR sculpting intervention effectively reduced negative emotions such as anxiety and tension (Z = 0.00, p < 0.001).

**Table 4:** Comparison of key measurements between control and experimental phases (N = 30).

Variable	Control phase Mean (SD)	Experimental Phase Mean (SD)	Test Method	Test Statistic	p-Value	Statistical Significance (p < 0.05)
GAD-7 (Anxiety Score)	6.90 (2.10)	4.10 (1.85)	Paired t-test	7.19	6.43e-08	Yes
PANAS+ (Positive Affect)	24.50 (2.31)	29.20 (2.82)	Paired t-test	-9.04	6.12e-10	Yes
PANAS- (Negative Affect)	20.10 (2.45)	16.45 (2.28)	Wilcoxon Signed-Rank Test	0.00	1.51e-05	Yes

### 3.2 Changes in GAD-7 (anxiety score)

To further analyze changes in anxiety levels, participants were categorized based on their initial GAD-7 scores (minimal/no anxiety, mild anxiety, moderate anxiety, and severe anxiety). Their anxiety reduction after the intervention was recorded. As shown in **Table 5**, individuals with mild and moderate anxiety exhibited a significant decrease in the experimental phase, indicating that the VR sculpting intervention primarily alleviated anxiety symptoms in these two groups. In contrast, individuals with minimal or no anxiety showed a smaller change, possibly because their baseline anxiety levels were already low, making the intervention effect less pronounced compared to the high-anxiety group.

**Table 5:** Changes in anxiety levels by group.

Anxiety Level	N	Experimental Phase GAD-7 Mean (SD)	Reduction in GAD-7	Intervention Effect Classification
Minimal or No Anxiety (0-4)	8	2.5 (1.2)	-1.5	Mild Improvement
Mild Anxiety (5-9)	14	3.8 (1.5)	-3.2	Significant Improvement
Moderate Anxiety (10-14)	4	6.1 (1.8)	-4.0	Significant Improvement
Severe Anxiety (15-21)	0	—	—	—

### 3.3 PANAS (positive and negative affect) analysis

Following the VR sculpting intervention, participants exhibited significant changes in emotional states. Positive affect (PANAS+) increased, while negative affect (PANAS-) decreased, demonstrating that VR sculpting not only alleviates anxiety but also enhances positive emotional experiences. As shown in **Table 6**, PANAS+ significantly increased in the experimental phase (t = -9.04, p < 0.001), indicating that the VR sculpting intervention effectively enhanced well-being, allowing participants to experience more positive emotions such as satisfaction and joy during the creative process. PANAS- significantly decreased in the experimental phase (Z = 0.00, p < 0.001), suggesting that the VR sculpting intervention significantly reduced negative emotions such as anxiety and tension.

**Table 6:** Changes in PANAS (positive and negative affect).

Variable	Control Phase Mean (SD)	Experimental Phase Mean (SD)	Test Method	Test Statistic	p-Value	Statistical Significance (p < 0.05)
PANAS+ (Positive Affect)	24.50 (2.31)	29.20 (2.82)	Paired t-test	-9.04	6.12e-10	Yes
PANAS- (Negative Affect)	20.10 (2.45)	16.45 (2.28)	Wilcoxon Signed-Rank Test	0.00	1.51e-05	Yes

### 3.4 HRV (heart rate variability) analysis

HRV serves as a key physiological measure of relaxation. The study found that HRV significantly increased after the intervention, suggesting that VR sculpting not only improves psychological well-being but also enhances physiological relaxation. As shown in **Table 7**, HRV significantly increased in the experimental phase (t = -7.20, p < 0.001), indicating that the VR sculpting intervention promoted physiological relaxation. Interviews revealed that many participants reported a strong sense of immersion during the VR sculpting process, allowing them to fully focus on their creation and reduce physiological tension.

**Table 7:** HRV changes.

Variable	Control Phase Mean (SD)	Experimental Phase Mean (SD)	Test Method	Test Statistic	p-Value	Statistical Significance (p < 0.05)
HRV (Heart Rate Variability)	38.70 (3.12)	45.60 (4.05)	Paired t-test	-7.20	4.21e-08	Yes

### 3.5 Summary of key findings

After the experimental phase, GAD-7 scores decreased significantly, indicating that VR sculpting exerts a notable effect on alleviating university students’ anxiety. PANAS+ (positive affect) rose significantly, while negative affect (PANAS–) declined markedly, suggesting that VR sculpting not only alleviates anxiety but also contributes to enhanced psychological well-being. Additionally, HRV increased significantly, revealing that VR sculpting promotes an effective state of physiological relaxation. Despite the overall impact, individual responses to anxiety relief and emotional enhancement varied—likely influenced by personal interest, familiarity with VR, and the creative experience. Future studies should consider incorporating standardized user experience and cybersickness scales to assess VR engagement and usability.

Overall, findings from this study demonstrate that VR sculpting, as a non-pharmacological intervention, yields substantial benefits in mitigating college students’ anxiety, elevating psychological well-being, and improving physiological relaxation. These results provide robust empirical support for employing art therapy—particularly VR-based methods—in campus mental health programs. Future research should further explore the long-term impact and underlying mechanisms of such interventions to enhance their generalizability and scalability.

## 4 Conclusion

This study aimed to assess the efficacy of “immersive sculpting” interventions on anxiety and psychological well-being among college students, employing GAD-7, PANAS, and heart rate variability (HRV) as multidimensional indicators. The results indicated that participants experienced a significant reduction in anxiety levels during the experimental phase compared to the control phase, along with notable increases in positive affect, decreases in negative affect, and marked improvements in HRV. These empirical findings offer new evidence supporting the application of art therapy in university settings and reinforce the unique value of non-pharmacological interventions for anxiety management and emotional regulation in college students.

Nonetheless, it is important to recognize that some participants reported only moderate or minimal improvement, which may be attributed to individual factors such as personal interest in art, prior creative experience, motivation for creation, or current psychological state. Additionally, the relatively short intervention period and limited sample size prevented an exhaustive examination of the intervention’s long-term effects. Moreover, the measurements primarily focused on anxiety, emotion, and HRV, lacking additional physiological or neuroscientific indicators (e.g., EEG, skin conductance) to delve deeper into potential mechanisms of change. These constraints suggest that future research should expand the sample size, lengthen the intervention and follow-up periods, and incorporate more diverse physiological or cognitive measures to elucidate the mechanisms underlying immersive sculpting in supporting college students’ mental health.

From a practical standpoint, building on this study’s findings, universities could adopt flexible approaches to integrate sculpting into art electives, mental health courses, or dorm-based clubs and organizations, providing a varied and engaging path for emotional self-regulation to a wider range of students. Existing research shows that art therapy can effectively improve the psychological state of students and foster positive emotions while lowering anxiety and depression. As an integral measure for campus mental health support, art therapy demonstrates high acceptability and notable efficacy in stress management and emotional regulation. Furthermore, collaboration among campus counseling centers, professional counselors, relevant educators, and external community resources could yield tailored intervention programs that address the diverse interests and requirements of different students. The combination of art therapy, individualized guidance, and ongoing follow-up can noticeably enhance intervention outcomes, expanding the effectiveness and sustainability of mental health initiatives in higher education.

In implementing art-based programs at universities, sculpting and other creative modalities not only supplement existing psychological counseling services, but also strengthen students’ self-esteem and resilience, thereby improving their capacity to cope with stress. Through consistent monitoring, periodic assessment, and personalized coaching, immersive sculpting could integrate with other art therapies to form a multifaceted on-campus mental health support system. Studies have shown that campus art therapy can elevate students’ sense of self-efficacy and provide an accessible, impactful pathway to self-help and personal growth in the face of academic pressures and future uncertainties. Consequently, incorporating sculpting and other art forms into collegiate mental health education not only enhances students’ psychological well-being, but also diversifies emotional management methods, fostering a more open and inclusive mental health culture.

## Data Availability

The original contributions presented in the study are included in the article/supplementary material. Further inquiries can be directed to the corresponding author.
